# Regulation of Protein Secretion Systems Mediated by Cyclic Diguanylate in Plant-Interacting Bacteria

**DOI:** 10.3389/fmicb.2019.01289

**Published:** 2019-06-12

**Authors:** Francisco Javier López-Baena, Jose María Vinardell, Carlos Medina

**Affiliations:** Departamento de Microbiología, Facultad de Biología, Universidad de Sevilla, Sevilla, Spain

**Keywords:** cyclic diguanylate, protein secretion systems, diguanylate cyclase, phosphodiesterase, adhesins, extracellular degradative enzymes, effector proteins, ATPase

## Abstract

The ubiquitous second messenger cyclic diguanylate (c-di-GMP) is involved in the regulation of different processes in bacteria. In phytopathogens, intracellular fluctuations in the concentration of this molecule contribute to the lifestyle switching from a motile and virulent stage to a sessile and biofilm-forming phase. Among the virulence mechanisms used by bacterial pathogens, different specific type secretion systems (TSSs) and the effector proteins that they translocate are included. Some of these TSS are conceived to suppress host immune responses during bacterial colonization. The modulation of the expression of secretion systems components and/or effector proteins can be influenced by c-di-GMP levels at transcriptional, translational, or post-translational levels and can take place directly by binding to specific or global regulators, or *via* transducer proteins. Different genera of plant-interacting bacteria have been analyzed to shed some light in the implications of c-di-GMP in the regulation of host plant colonization through protein secretion systems. Expression of (1) adhesins secreted by Type 1 secretion systems to bind the host plant in *Pectobacterium* (formerly *Erwinia*) and some beneficial *Pseudomonas* strains; (2) catalytic exoproteins delivered by Type 2 secretion systems to break plant cell wall in *Dickeya*; (3) effectors secreted by Type 3 secretion systems to suppress plant immunity in *Xanthomonas*; or (4) the activity of Type 6 secretion systems to export an ATPase in *Pseudomonas*, are finely tuned by c-di-GMP levels. In this minireview, we summarize the knowledge available about the implications of c-di-GMP in the regulation of protein secretion in different plant-interacting bacteria.

**Topic:** Secretion systems and effector proteins of phytopathogenic and beneficial bacteria regulated by NSM.

## Introduction

Bacteria have developed complex regulatory signaling circuits where small molecules serve as messengers that confer abilities to adapt to changing environments. Among these molecules, cyclic diguanylate (c-di-GMP) has received grand attention lately due to the huge variety of reactions in which it is involved, especially regarding eukaryotic cell-interacting bacteria. The intracellular concentration of c-di-GMP is modulated by the action of diguanylate cyclases (DGCs) or phosphodiesterases (PDEs), enzymes that synthesize c-di-GMP by condensation of two GTPs or degrade it, respectively. DGCs contain a conserved GGDEF domain, while PDEs present either EAL or HD-GYP motifs to generate linear dinucleotide pGpG or GMP, respectively (Ryan et al., 2007). Alterations in c-di-GMP concentration affect signaling networks that, in many cases, are responsible for lifestyle switching. Elevated concentrations of c-di-GMP by the action of DGCs are associated with biofilm formation and a sessile life stage related to pathogenic chronic infections, whereas reduced concentrations of c-di-GMP provoked by PDEs are found in motile bacteria responsible for acute infection processes (Moscoso et al., 2011). Curiously, the case of the olive pathogen *Xylella fastidiosa* seems to be the opposite, revealing that each bacterium has integrated the role of c-di-GMP according to its own colonization process (Chatterjee et al., 2010). These signaling networks are linked with external stimuli that transmit input signals to regulatory proteins that integrate c-di-GMP metabolism into global regulatory circuits. Thus, responding to determined environmental cues, bacteria can increase the production of extracellular polysaccharides required for biofilm formation and reduce the synthesis of virulence factors like toxins and/or effector proteins delivered by distinct secretory systems, or vice-versa.

The c-di-GMP regulation processes are mediated by its interaction with specific receptors, but only a few known proteins containing canonical c-di-GMP binding motifs have been identified so far. In addition, as the intracellular concentration of c-di-GMP seems to be essential in these processes, the use of synthetic systems able to artificially alter this concentration becomes desirable (Pérez-Mendoza et al., 2014). Since the number of c-di-GMP metabolizing enzymes is increasing following the massive genomes sequencing era, an effort must be done to identify c-di-GMP binding sensors or receptors. Different possible c-di-GMP effector mechanisms have been proposed: sensor proteins including c-di-GMP binding domains as PilZ that interact with regulatory or metabolizing enzymes, allosteric binding domains surrounding c-di-GMP catalytic motifs, regulatory proteins that bind c-di-GMP by themselves, or RNA riboswitches (Jenal and Malone, 2006). c-di-GMP has been suggested to be involved in regulatory processes at transcriptional, post-transcriptional, and post-translational levels (Hickman and Harwood, 2008; Sudarsan et al., 2008; Newell et al., 2011) in a multi-tier way. Moreover, c-di-GMP is also directly implicated in the translocation process in almost all described protein secretion systems present in Gram-negative bacteria (Trampari et al., 2015). In this minireview, we summarize the effects of c-di-GMP over the regulatory events governing protein secretion in distinct beneficial or pathogenic plant bacteria (Table 1). We do apologize for all the information omitted, fundamentally due to the limited space available.

**Table 1:** A compendium of the involvement of c-di-GMP in secretion systems of Gram-negative bacteria.

Secretion system	Secreted protein	Role	Bacterial specie	DGC or PDE	Reference
T1SS	ECA3266	Secretion of a proteinaceous multi-repeat adhesion (MRP)	*Pectobacterium atrosepticum*	ECA3270 ECA3271	Pérez-Mendoza et al., 2011
T1SS	LapA, LapF	Adhesion	*Pseudomonas fluorescens* *P. putida*	RapA BifA	Martínez-Gil et al., 2014; Monds et al., 2007; Xiao et al., 2016
T1SS	BpfA	Adhesion	*Shewanella putrefaciens*	DosD	Wu et al., 2013
T2SS	Peptidases or Aminopeptidases	Virulence	*Xanthomonas oryzae*	DgcA	Su et al., 2016
T2SS	Pel N,L,X	Pectate lyase	*Dickeya dadantii*	Related to GacA/S	Hassan et al., 2013
T2SS	Pel D	Pectate lyase	*D. dadantii*	EcpC, EGcpB, GcpA	Yuan et al., 2018
T2SS	Proteases	Virulence	*P. aeruginosa*	MorA^a^	Ravichandran et al., 2015
T4P	Pil A, pilins	Motility	*Myxococcus xanthus*	DmxA , SgmT	Skotnicka et al., 2016
T4P	T4P genes	Motility	*P. aeruginosa*	SadC^b^	Francis et al., 2017
T3SS	hrp genes	Virulence	*Xanthomonas oryzae* (Xoo)	PdeR , GdpX1	Yang et al., 2012, 2016
T3SS	hrp genes	Virulence	*Xanthomonas oryzae* (Xoc)	XOC_2335 XOC_2393	Wei et al., 2016
T3SS	Proline Iminopeptidase (PIP)	Virulence	*Xanthomonas campestris* (Xcc)	Related to PIP	Kan et al., 2018
T3SS	*hrp* genes	Virulence	*P. syringae*	Chp8	Engl et al., 2014
T3SS	*hrp* genes	Virulence	*D. dadantii*	EGcpB, EcpC CsrD, GcpA	Yi et al., 2010 Wu et al., 2014; Yuan et al., 2015, 2018
T3SS	*hrpA*	Virulence	*Erwinia* *amylovora*	EdcC and EdcE	Edmunds et al., 2013
T5SS	CdrA^d^	Adhesion	*P. aeruginosa*	Not defined	Cooley et al., 2016
T6SS	Hcp1	Virulence	*P. aeruginosa* *P. savastanoi*	DgcP	Aragon et al., 2015
T6SS	Hcp1	Virulence	*P. putida*	BifA	Wang et al., 2017
T6SS	Hcp1	Virulence	*P. aeruginosa*	WspR^c^ , PA2133	Moscoso et al., 2011
T6SS	Hcp1	Quorum sensing	*Vibrio alginolyticus*	Related to PppA	Cheng et al., 2013

a MorA is also present in some plant-interacting *Pseudomonas* species such as *P. syringae* and *P. fluorescens* (Prada-Ramírez et al., 2016; Liu et al., 2018), but its involvement in regulation of T2SS has only been demonstrated in *P. aeruginosa* so far.

b Although SadC is present in some plant-interacting *Pseudomonas* species such as *P. fluorescens* and *P. putida* (Martínez-Granero et al., 2014), its involvement in regulation of T4P has only been demonstrated in *P. aeruginosa* so far.

c WspR has also been found in some plant-interacting *Pseudomonas* species such as *P. fluorescens, P. putida*, *P. syringae,* and *P. protegens* (Martínez-Granero et al., 2014; Pfeilmeier et al., 2016), but its involvement in regulation of T6SS has only been demonstrated in *P. aeruginosa* so far.

d CdrA-like proteins participating in adhesion have been identified in diverse *Proteobacteria* such as *Rhizobium tropici* CIAT 899 and the *Burkholderiales* bacterium JOSHI_001 (Cooley et al., 2016).

## Type One Secretion System

The type one secretion system (T1SS) can secrete a high diversity of substrates, from small toxins to large adhesins, in many Gram-negative bacteria (Smith et al., 2018). The T1SS is composed by an ATP-binding cassette (ABC) transporter anchored to the inner membrane linked to a membrane fusion protein that bridges the inner membrane to the outer membrane component, a TolC-like pore (Green and Mecsas, 2016). Protein translocation may occur as a one-step mechanism, such as for the toxins HlyA of *Escherichia coli* (Thomas et al., 2014) or CyaA of *Bordetella pertussis* (Novak et al., 2017), or *via* a periplasmic intermediate, such for LapA and F adhesins of *Pseudomonas putida* KT2440 and *P. kilonensis* F113 (formerly *Pseudomonas fluorescens*) (Smith et al., 2018).

**Figure 1:**
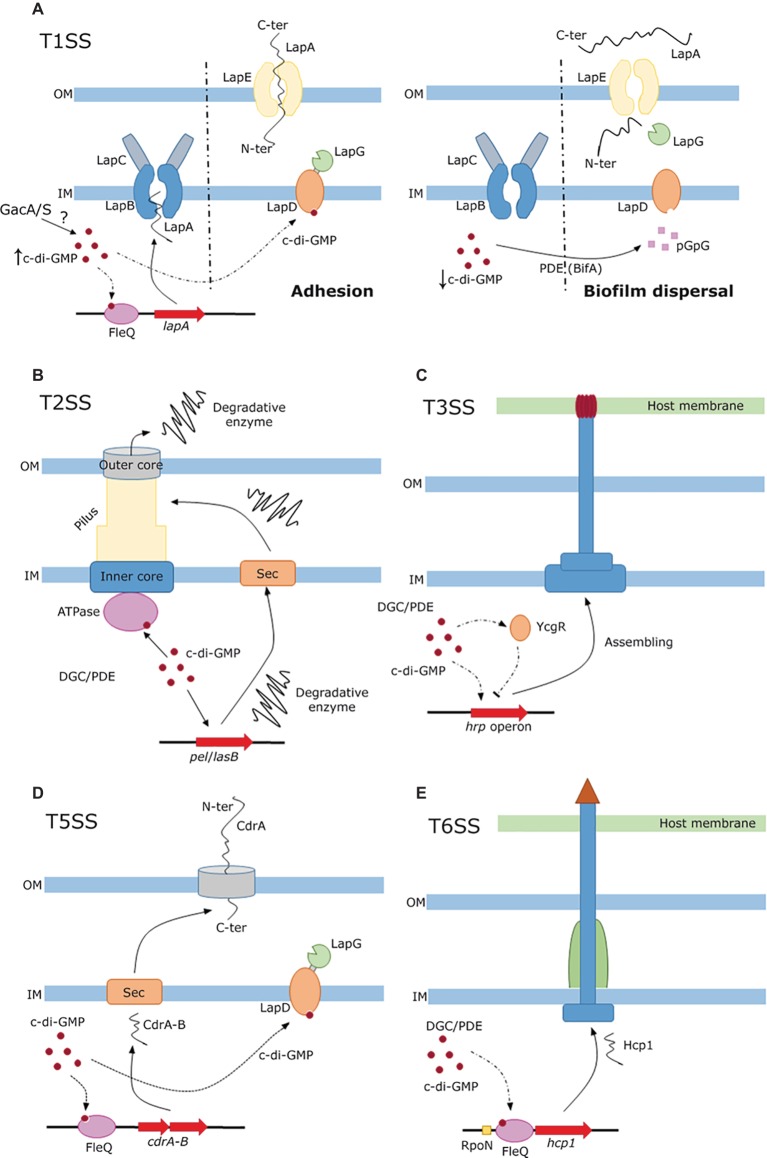
c-di-GMP-dependent regulation of protein secretion through different secretion systems. **(A)** Regulation of LapA secretion through the T1SS and its role in biofilm formation in pseudomonads. High concentration of c-di-GMP acts at two levels: (1) c-di-GMP is sensed by FleQ, which activates the transcription and subsequent secretion of LapA *via* the inner membrane complex (LapB,C) to the outer membrane pore LapE resulting in bacterial adhesion, and (2) c-di-GMP activates LapD that sequesters the specific protease LapG (left). Upon BifA action, c-di-GMP concentration diminishes and LapD releases LapG which cleaves LapA that is relief to the media inducing biofilm dispersal (right). **(B)** T2SS mediated translocation of extracellular degradative enzymes. Low c-di-GMP concentration participates in the transcription and sec-dependent secretion of several degradative enzymes, which are pushed out to the extracellular milieu by the c-di-GMP mediated activation of the ATPase machinery at the inner membrane. **(C)** Partial involvement of c-di-GMP regulation network of T3SS mediated secretion in Dickeya. c-di-GMP modulates the expression of the *hrp* operon, *via* the sensing protein YcgR and in an unclear manner through the RpoN sigma factor. A detailed description of the network is provided in the text and in Yuan et al. (2015). **(D)** T5SS and Sec-dependent secretion of CdrA. The adhesin CdrA and its T5SS cognate transporter CdrB are secreted to the periplasm prior to the inclusion in the outer membrane of the latter. As in the case of LapA secretion, c-di-GMP participates in its regulation at transcriptional level *via* FleQ and post-translationally by binding to the protease sequester LapD. **(E)** Role of c-di-GMP in the regulation of translocation *via* T6SS. High levels of c-di-GMP upregulates the T6SS and promotes the secretion of the hallmark protein Hcp1. Arrowheads and bar-heads edges represent activation or inhibition of expression respectively.

The regulation of LapA and LapF synthesis and secretion has been extensively analyzed in plant beneficial pseudomonads (Monds et al., 2007; Martínez-Gil et al., 2014; Jiménez-Fernández et al., 2016; Xiao et al., 2016), and they are finely tuned by several components of a regulatory network orchestrated by c-di-GMP. The success of bacterial thriving in different natural niches is favored by the capacity to attach to surfaces and establish biofilms, where the adhesins LapA and LapF play important roles at distinct stages. Secretion of Lap adhesins starts in response to an undetermined environmental signal that leads to autophosphorylation of the GacS sensor, which transmits its phosphate group to the transcriptional regulator GacA. GacA activates, directly or through other regulators, several genes coding for small regulatory RNAs. This activation in turn triggers a regulatory cascade that primes transcription of different genes. The role of regulator FleQ and the RpoS sigma factor are remarkable due to their connection with LapA and LapF secretion. Once the GacS/GacA system initiates the transduction of the environmental cue, the level of c-di-GMP increases by still undetermined mechanisms inside the bacterium and binds to the transcriptional activator FleQ, finally resulting in the synthesis and secretion of LapA. Translocation of LapA occurs from its C-terminus that passes through the ABC transporter LapB linked to the membrane fusion protein LapC, which channels LapA to the outer membrane pore LapE, inducing the initial and irreversible attachment of the bacteria to a surface in a first step of biofilm formation (Figure 1A-left). During biofilm development, RpoS, which is activated during the stationary phase, contributes to the synthesis and secretion of LapF (*via* another ABC transporter named *lapHIJ*) that serves as a cell-to-cell linker that maintains the structure of the nascent microcolony (Martínez-Gil et al., 2014). Under nutrient starvation, the biofilm initiates a regulated dispersal process that includes the regulatory role of c-di-GMP. While the biofilm is being formed, the regulatory protein LapD binds c-di-GMP inducing the sequestration of the periplasmic protease LapG. Upon conditions of biofilm dispersal, the concentration of c-di-GMP diminishes by distinct PDEs as BifA and therefore LapD releases LapG that cleaves a specific site of the adhesin LapA, releasing it to the medium by the outer membrane pore LapE, causing the biofilm dispersal (Figure 1A-right). This complex secretion process of adhesins is shared by other bacteria such as *Bordetella bronchiseptica* (Ambrosis et al., 2016), *Pectobacterium atrosepticum*, or *Citrobacter youngae* (Pérez-Mendoza et al., 2011).

## Type Two Secretion System and Type Four Pili

The type two secretion system (T2SS) has been identified in a huge number of eukaryote-interacting Gram-negative bacteria. This secretion system translocates folded proteins, previously secreted to the periplasm by Sec or Tat pathways, across the outer membrane to the extracellular milieu. A plethora of exoproteins are secreted, including extracellular degradative enzymes with diverse biological functions, as well as toxins, which collectively contribute to bacterial pathogenesis and/or virulence (Korotkov et al., 2012).

T2SS spans both membranes of Gram-negative bacteria and is composed of 12–15 proteins conforming a sophisticated machinery that comprises four separate platforms attending to its function and location. The inner membrane complex is composed of different proteins and serves as a nexus among the other components of the system, namely, the outer membrane core (formed by a polymerized protein known as secretin that generates a pore in the outer membrane), the cytoplasmic ATPase (which energizes the system), and the periplasmic (pseudo)pilus. This last component is composed by an abundant major pilin and other minor pilins whose assembling and disassembling works as a piston to impulse proteins outside the bacteria (Korotkov et al., 2012).

This particular architecture is analog to the type four pilus (T4P), responsible for the twitching motility exhibited by several pathogenic bacteria (Burrows, 2012). T2SS and T4P functionality is also similar and the energy required for the extension and retraction of minor pilins in both cases comes from the cytoplasmic ATPase along the inner membrane component (Hazes and Frost, 2008). Occasionally, the T4P can mediate the secretion of some virulence factors in *Vibrio cholerae* (Kirn et al., 2003).

It has been proposed that the major pilin of T2SS and T4P is secreted *via* Sec-pathway and integrates into the inner membrane (Francetic et al., 2007). The involvement of c-di-GMP in the regulation of T4P dependent motility is subjected to study in several plant-interacting bacteria since, once colonized the surface of a plant (leaves or roots), bacteria migrate across such surfaces in a pili or flagella dependent manner toward a determined entry site (Pfeilmeier et al., 2016). This motility is important in *Lysobacter enzymogenes*, a soil bacteria used in biocontrol against plant crop fungal pathogens that directly attach to the hypha through T4P, producing subsequently extracellular lytic enzymes to colonize the fungus. The genes involved in the biosynthesis of T4P are upregulated by Clp, a global transcriptional regulator present in a variety of bacteria as *Lysobacter* or *Xanthomonas* that binds c-di-GMP to modulate virulence by control gene expression (Chin et al., 2010; Wang et al., 2014; Yang et al., 2015). In *Myxococcus xanthus*, increased concentrations of c-di-GMP diminish *pilA* transcription (T4P major pilin), hence reducing T4P assembly and twitching motility (Skotnicka et al., 2016). In *P. aeruginosa,* activation of the diguanylate cyclase SadC by a complex regulation promotes downregulation of T4P genes (reviewed in Francis et al., 2017).

c-di-GMP can also bind to a newly described motif of T2SS- and T4P-associated ATPases (MshE in *V*. *cholerae* and its homolog XpsE in *X. campestris*; Roelofs et al., 2015; Wang et al., 2016). Although the exact mechanism of regulation remains to be elucidated, it has been proposed that c-di-GMP binding to the ATPase releases energy that could enhance the export of MshA pili monomers or virulence factors (Figure 1B).

The protease secretion across T2SS is also negatively affected at post-translational level by the action of MorA in *P. aeruginosa* (Ravichandran et al., 2015). This protein, albeit showing PDE activity is mainly a DGC, and it is conserved among diverse *pseudomonds* as such as *P. syringae* (Prada-Ramírez et al., 2016) or *P. fluorescens* (Liu et al., 2018).

In *Stenotrophomonas maltophilia*, present in plant rhizosphere and able to promote plant growth, the secretion of an extracellular protease is dependent on temperature *via* a two-component system that modifies the intracellular concentration of c-di-GMP. This alteration is sensed by Clp that promotes the production of the protease (Wang et al., 2018).

The *Dickeya dadantii* pectate lyase (Pel) is a cell-wall degrading enzyme released by the T2SS that causes soft-root diseases in plants (Expert et al., 2018). The biosynthesis of this enzyme is negatively regulated by high levels of c-di-GMP *via* a complex regulatory circuit that involves the regulatory RNA RsmB, the PDE CsrD, and the periplasmic glucans OpgGH (Wu et al., 2014).

## Type Three Secretion System

This is probably the best characterized protein injection apparatus synthesized by bacteria to deliver proteins into the cytosol of eukaryotic cells. The Type Three Secretion System (T3SS) is widespread in nature in both pathogens and symbionts and although the secretory machinery is well conserved, the set of effector proteins delivered is specific for each strain (Galán et al., 2014). T3SS structure is similar to a syringe and consists of three main components. The basal complex spans both inner and outer membranes and is composed of at least 15 proteins assembled as several rings surrounding a central rod. The needle conforms a hollow tube through which the unfolded protein is secreted. Finally, the outer translocon, essential to recognize the host cell membrane, forms a pore for effector delivery.

T3SS is the bacterial secretion system in which the implication of c-di-GMP has been most extensively studied. *Xanthomonas oryzae* pv. *oryzae* (*Xoo*) is the causal agent of bacterial leaf blight of rice, one of the most dramatic rice diseases worldwide. As in other phytopathogens, regulation of its T3SS-mediated virulence is highly complex, and the implication of c-di-GMP signaling, currently under study, involves both c-di-GMP metabolizing enzymes and receptors. The intracellular concentration of c-di-GMP is modulated by PDEs such as PdeR (Yang et al., 2012) and DGCs such as GdpX1 (Yang et al., 2016) whose opposite actions are involved in the regulation of the pathogenicity (*hrp*) gene cluster that controls T3SS expression. Consistently with other bacteria, a high c-di-GMP concentration provoked by the action of GdpX1 negatively regulates the expression of T3SS-related genes. Afterward, c-di-GMP should be sensed by receptor proteins such as FilP, which interacts with the PilZ protein PXO_02715 and regulates the expression of the T3SS at the transcriptional level (Yang et al., 2014).

Some effector proteins secreted by the T3SS are related with the metabolism of c-di-GMP. *X. campestris* pv*. campestris* (*Xcc*) is a pathogenic bacterium responsible for the black root disease in cruciferous plants. Proline iminopeptidase (PIP) is a secreted effector protein conserved in many plant pathogens that have shown a dual effect on *Xcc*. In one hand, its disruption decreases c-di-GMP levels in the bacteria directly or indirectly *via* an unknown target, while when translocated through the T3SS into the plant contributes to suppression of plant immunity (Kan et al., 2018).

Biosynthesis of the T3SS is also regulated by c-di-GMP through a sophisticated regulatory network in *D. dadantii*, the causal agent of soft root disease in many economically important crops (Yi et al., 2010; Wu et al., 2014; Yuan et al., 2015, 2018). The alternative sigma factor HrlP drive the expression of the *hrp* operon that encodes structural components and effector proteins. Regulation of *hrlP* occurs at a transcriptional level *via* the additive action of the RpoN sigma factor and its enhancer HrpS, whose expression is controlled by a two-component system (HrpX/Y), and post-transcriptionally by the regulatory sRNA-binding protein RsmA, which contributes to the declination of *hrlP* mRNA. RsmA is sequestered by the sRNA *rsmB*, controlled by the two-component system GacS/A, preventing its *hrlP* mRNA degradative action. Furthermore, high concentrations of c-di-GMP generated in the PDE mutants EcpC and EGcpB reduce the transcription of *hrlP via* RpoN in an unclear manner. In a higher tier, the flagellar master regulator FlhDC controls T3SS expression hierarchically by three distinct pathways. (1) FlhDC drives the expression of the alternative sigma factor FliA that regulates at a transcriptional level the c-di-GMP sensing protein YcgR. This protein, upon elevated concentration of c-di-GMP, negatively regulates the expression of the T3SS (Figure 1C). (2) FlhDC and FliA post-transcriptionally control the regulatory sRNA *rsmB* in opposite ways. Finally, (3) FlhDC tunes the expression of the PDE EcpC, which reduces the concentration of c-di-GMP resulting in the activation of T3SS *via* RpoN-*hrlP* regulation. The relationship of some of these components with the Pectate lyase production is detailed in T2SS section (Wu et al., 2014).

## Type Five Secretion System

This system is confined to the outer membrane and translocates proteins, previously secreted by the Sec pathway, from the periplasm to the extracellular medium. There are several kinds of type five secretion system (T5SS): autotransporters (Type 5a), two-partner secretion system (Type 5b), and chaperone assisted secretion (Gunasinghe et al., 2017). Among the substrates secreted, proteins playing important roles in bacterial pathogenesis are remarkable. It has been recently described a two-partner system in *P. aeruginosa*, CdrA-B, whose secretion is subjected to c-di-GMP regulation (Cooley et al., 2016). CdrA acts as an adhesion-like protein secreted to the periplasm by the Sec pathway and anchored by its C-terminal tail to the outer membrane by its cognate transporter CdrB. CdrA, like LapA, is released to the extracellular space by LapG proteolytic cleavage. Again, c-di-GMP regulation acts at transcriptional and post-translational levels since the transcription of the *cdrA-B* operon is promoted by FleQ activated by elevated concentrations of c-di-GMP, while c-di-GMP activates LapD hence inhibiting LapG proteolytic activity (Figure 1D). Only when c-di-GMP levels diminish, LapG is liberated and releases CdrA to the extracellular milieu.

Interestingly, despite the similarity that the regulation of this system shares with the secretion of LapA in *P. putida*, these two adhesins have not homology while LapD-G system does. This observation could serve to identify putative two-partner secretion systems under the control of LapG orthologs as is the case of *Burkholderiales* bacterium JOSHI_001 or the rhizobial strain *Rhizobium tropici* CIAT 899 (Cooley et al., 2016).

## Type Six Secretion System

This secretion system has been recently described in bacteria and acts as a protein injector into eukaryotic hosts or other bacteria (Mougous et al., 2006). Some authors hypothesize, due to its structural similarities to a phage tail, that an ancestor bacterium acquired some structural components of a tailed bacteriophage and lately used it to develop a secretory system to eject proteins outside the cell (Leiman et al., 2009; Filloux, 2013). Computational predictions estimate that this system is present in over 25% of bacterial species (Basler et al., 2012) and contributes to virulence of some bacterial pathogens since it is associated to biofilm stages. In *P. putida* three type six secretion systems (T6SSs) have been described (K1–K3). K1-T6SS functions as an antibacterial device able to eradicate phytopathogens like *X. campestris* on *Nicotiana benthamiana* leaves (Bernal et al., 2017).

The overexpression of the PDE BifA has revealed the implication of c-di-GMP in the expression of the secreted protein Hcp1, which is a hallmark of T6SS activity (Wang et al., 2017). Thus, when the levels of c-di-GMP are synthetically reduced, the transcriptional regulator FleQ in concert with the specific sigma factor RpoN negatively regulates the expression of Hcp1 and therefore T6SS assembling in *P. putida*. On the other hand, when c-di-GMP levels are increased by overexpression of the DGC DgcP, the T6SS is upregulated as detected by the expression and secretion of Hcp1 (Figure 1E). This observation correlates with the proposed negative control of motility and positive control of biofilm formation by de DGC DgcP (conserved in pseudomonads) after its deletion of its encoding gene in the olive tree pathogen *P. savastanoi* (Aragon et al., 2015).

The T6SS has been associated with a sessile and biofilm stage of plant interacting bacteria, while T3SS is expressed in a more virulent and motile phase. The differential activation of each secretion system and the corresponding lifestyle switching respond to environmental signals that are sensed by the two-component regulatory system GacS/GacA connected to the RetS/LadS kinases present in a variety of pseudomonads as *P. fluorescens* (Workentine et al., 2009) or *P. syringae* (Records and Gross, 2010). Briefly, under determined environmental signals, the sensor LadS activates the GacA/GacS signaling cascade, and the transcriptional regulator RsmA is sequestered by the small RNAs RsmY/RsmZ, leading to biofilm formation and T6SS expression. Contrarily, upon environmental changing conditions, the sensor RetS binds GacS, avoiding GacA activation what results in the activation of RsmA, which in turn promotes the activation of T3SS and bacterial motility and represses genes related to biofilm formation and to the biosynthesis of antifungal secondary metabolites as in the case of *P. fluorescens* (Zhang et al., 2015). This lifestyle switching has also been associated to fluctuations in c-di-GMP levels in the model *P. aeruginosa*, in which the DGC WspR increases the levels of c-di-GMP favoring T6SS and repressing T3SS, whereas the PDE PA2133 acts opposite (Moscoso et al., 2011).

## Concluding Remarks

Research on c-di-GMP signaling was initiated during the study of cellulose biosynthesis in *Gluconacetobacter xylinus*, which primed the investigation of its implication in the regulation of various cellular aspects in addition to exopolysaccharide synthesis, such as biofilm formation, bacterial motility, or protein secretion. The efforts of numerous researchers on these topics have led to the assumption that the role of this nucleotidic second messenger is critical for driving a bacterial lifestyle switching from motile to sessile in eukaryote-hosts interacting bacteria through the activation of different secretion systems. These bacteria have two main stages, a motile and virulent phase characterized by the activation of mechanisms involved in the production and secretion of virulence factors that participate in the invasion of the host, and a sessile stage in which bacteria promote biofilm formation by means of the production and secretion of different extracellular polysaccharides and other secondary metabolites.

Environmental cues are sensed by different receptors that, directly or indirectly, modify the intracellular concentration of c-di-GMP, resulting in the activation of a specific secretion system and in a lifestyle switching. The virulent and motile phase are enhanced by low levels of c-di-GMP that participate in the expression of virulence factors mainly secreted through the T2SS and/or the T3SS, which upon this condition is mostly upregulated. On the opposite, the sessile lifestyle is induced by high levels of c-di-GMP that trigger the production and secretion of adhesins *via* T1SS and T5SS and upregulate the T6SS, which is also related to biofilm formation.

Nevertheless, we only have some pieces of such a puzzle and further research is required to lighten the direct implication of c-di-GMP in the regulation of virulence and bacterial host colonization. Whereas the number of identified c-di-GMP metabolizing enzymes increases progressively with the massive genome sequencing, the number of known c-di-GMP interacting molecules (proteins or RNAs) is still poor. Novel technologies and implementation of current ones will help to define still unknown c-di-GMP interacting motifs and thus unravel the regulatory networks that lead to protein secretion in Gram-negative bacteria.

## Author Contributions

CM conducted the main work. FL-B designed and developed the figure and table and discussed the body of the paper. JV critically reviewed the manuscript and contributed to the final design of the manuscript.

### Conflict of Interest Statement

The authors declare that the research was conducted in the absence of any commercial or financial relationships that could be construed as a potential conflict of interest.

## Abbreviations

c-di-GMP: 3′, 5′-Cyclic diguanylic acid
DGC: Diguanylate cyclase
GMP: Guanosine monophosphate
GTP: Guanosine triphosphate
PDE: Phosphodiesterase
pGpG: Linear dinucleotide 5-phosphoguanylyl-(3′,5′)-guanosine
